# Ultra-broadband coherent open-path spectroscopy for multi-gas monitoring in wastewater treatment

**DOI:** 10.1016/j.ese.2025.100554

**Published:** 2025-03-17

**Authors:** Roderik Krebbers, Kees van Kempen, Yueyu Lin, Joris Meurs, Lisanne Hendriks, Ralf Aben, José R. Paranaiba, Christian Fritz, Annelies J. Veraart, Amir Khodabakhsh, Simona M. Cristescu

**Affiliations:** a Life Science Trace Detection Laboratory, Department of Analytical Chemistry & Chemometrics, Institute for Molecules and Materials, Radboud University, Heyendaalseweg 135, AJ Nijmegen, 6525, the Netherlands; b Department of Ecology, Radboud Institute for Biological and Environmental Science, Radboud University, Heyendaalseweg 135, AJ Nijmegen, 6525, the Netherlands

**Keywords:** Infrared spectroscopy, Greenhouse gas emissions, Fourier transform spectroscopy, Methane, Nitrous oxide, Ammonia

## Abstract

Wastewater treatment plants significantly contribute to greenhouse gas emissions, including nitrous oxide (N_2_O), carbon dioxide (CO_2_), and methane (CH_4_). Current methods to measure these emissions typically target specific molecular compounds, providing limited scope and potentially incomplete emissions profiles. Here, we show an innovative ultra-broadband coherent open-path spectroscopy (COPS) system capable of simultaneously monitoring multiple greenhouse gases. This novel approach combines Fourier transform spectroscopy with a coherent, ultra-broadband mid-infrared light source spanning 2–11.5 μm at approximately 3 W power. Positioned above an aeration tank, the COPS system selectively detected absorption signatures for CH_4_, CO_2_, N_2_O, ammonia (NH_3_), carbon monoxide (CO), and water vapor (H_2_O), enabling real-time, path-integrated concentration measurements with a temporal resolution of 40 s. Elevated concentrations of CH_4_ and CO_2_ were clearly identified within emission plumes traversing the beam path above the aeration tank. Additionally, CH_4_ emission patterns closely tracked variations in ammonium loading from incoming wastewater, whereas CO_2_ emissions correlated strongly with oxygen concentrations introduced during aeration. Measurements of N_2_O, NH_3_, and CO were stable and aligned closely with traditional point-based measurements from commercial gas analyzers. Our findings demonstrate that COPS offers a robust, comprehensive solution for the simultaneous real-time monitoring of multiple gases in complex and heterogeneous emission environments. This capability significantly enhances atmospheric and industrial emission assessments, potentially transforming the approach to emissions quantification and environmental management.

## Highlights

• Gas emissions from wastewater treatment plants (WWTPs) are challenging to monitor due to their heterogeneous nature.
• A novel coherent open-path spectroscopy (COPS) system was deployed for real-time gas monitoring at a WWTP.
• The system has unprecedented spectral coverage and resolution (2–11.5 μm, 0.1 cm^−1^) for simultaneous multi-gas detection.
• CH_4_ and CO_2_ emissions were detected and linked to specific operational processes within the plant.
• The system also continuously monitored N_2_O, NH_3_, CO, and H_2_O concentrations.

## Graphical abstract

**Figure:**
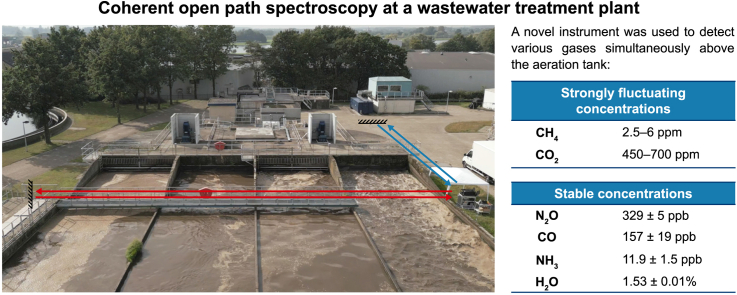

## 1 Introduction

Wastewater treatment plants (WWTPs) are known to contribute to greenhouse gas (GHG) emissions of carbon dioxide (CO_2_), methane (CH_4_), and nitrous oxide (N_2_O) [1,2]. However, only a few GHG emission datasets with sufficient spatial and temporal coverage are available from WWTPs. This limits the feasibility of modeling approaches, especially with the high heterogeneity that the current datasets show [3,4]. The best currently available practical methods for estimating GHG emissions revolve around two approaches: point sampling and open-path (also known as free-path) spectroscopy [5,6].

Point sampling measurements for GHG emission estimates at WWTPs can be taken via discrete samples from surface waters and the atmosphere combined with (often highly uncertain) estimates of air-water gas exchange [7,8], or by collecting off-gas from covered WWTP components [9]. Discrete sampling methods are limited by their temporal and spatial resolution: their sampling frequency is low compared to continuous monitoring techniques, and the spatial coverage is limited to specific points within the study area. Moreover, the potential contribution of microbubbles is excluded, which may be particularly important in WWTPs where aeration takes place [10,11].

Another point sampling approach is to use open and closed floating flux chambers, usually combined with online GHG analyzers (e.g., off-axis integrated cavity output spectrometers or cavity ring-down spectrometers) [12].

Flux chamber measurements offer snapshots of flux estimates and can be ideal for analyzing small-scale variations in GHG fluxes within aquatic ecosystems. However, flux chamber measurements face operational difficulties in the fast-flowing waters of, e.g., WWTPs, and the presence of the chamber itself can challenge the assumption that measured fluxes represent those outside of the chamber, for example, due to artificially enhanced within-chamber turbulence [[13], [14], [15]]. Moreover, both discrete water or gas sampling and flux chamber methods are currently labor-intensive and cover only a small surface area, which is a problem in systems with significant spatial heterogeneity.

Continuous spatiotemporal measurements with open-path spectroscopic techniques overcome the limitations of point sampling approaches. These techniques send a light beam over a free path through the area of interest to interact directly with the gas. This contrasts with point measurement techniques, where gas samples are captured in a gas cell for interaction with the light. Open-path techniques offer non-invasive, *in situ*, stand-off, and real-time detection of gas species, providing faster measurements and avoiding sampling complications with gases that are difficult to sample in gas cells due to wall-adhesion effects (such as ammonia (NH_3_)).

Differential optical absorption spectroscopy (DOAS) is a classical technique in open-path spectroscopy [[16], [17], [18]] using sunlight or a thermal source (usually in ultraviolet/visible spectrum) for gas detection (e.g. of GHGs and nitrogen oxides) typically in combination with a Fourier transform spectrometer (FTS) or grating-based spectrometer. As the brightness of the thermal source is limited, DOAS needs a long measurement time, provides a low spectral resolution, and thus results in limited applicability [19].

Coherent open-path spectroscopy (COPS) replaces thermal incoherent sources in DOAS systems with spatially and/or temporally coherent sources. These sources can be optimized for the near- (1–2.5 μm) and especially the mid-infrared (MIR) spectral range (2.5–20 μm) where most of the interesting gases present the strongest molecular absorption features. Dual-comb spectroscopy (DCS) systems are frequently used for COPS-based gas sensing, operating mostly in the near-infrared (NIR) spectral range [[20], [21], [22], [23], [24], [25], [26]]. Even though the technology for this spectral range is mature, gas sensing in NIR mostly relies on (weaker) overtone vibrational bands of molecular species, resulting in a limited number of detectable species and low detection sensitivities. More recently, DCS COPS systems have been employed in the MIR, however, either not extending further than 5 μm [[27], [28], [29], [30], [31]] or exclusively covering the region around 10 μm [32].

High-power supercontinuum (SC) sources are a compelling choice for COPS systems, given their high brightness, broad spectral coverage, and spatial coherence facilitating transmission over long distances and shorter measurement times [33]. In recent years, there have been significant developments in their availability for the MIR spectral region [34,35]. Current state-of-the-art SC sources achieve broad spectral coverage in MIR via nonlinear effects in special fibers [[36], [37], [38], [39]] or intrapulse difference frequency generation (IDFG) in nonlinear crystals [[40], [41], [42]]. With the exceptional spectral coverage of state-of-the-art SC sources, it is possible to cover the strongest, fundamental rovibrational bands of different molecular species (e.g., hydrocarbons, oxides, and small organic molecules) in the MIR spectral range from 2 to 11.5 μm [42], enabling multispecies detection of these molecules with high sensitivity.

Here, we present the results of the first field deployment of a COPS system with unprecedented spectral coverage (2–11.5 μm) in the MIR based on IDFG, yielding high detection sensitivities for a broad range of molecules. The system was deployed at a WWTP to characterize the gases present above the aeration tank. The results of the COPS system were compared to state-of-the-art commercial GHG analyzers, which measured the concentrations of GHGs in the emission plume from the aeration tank.

## 2 Materials and methods

### 2.1 The COPS system: a platform for simultaneous detection of greenhouse and polluting gases

Coherent open-path spectroscopy is a unique technique that combines laser-based gas spectroscopy with ultra-broadband spectral coverages, enabling sensitive measurements with a high dynamic range for a great variety of different gases simultaneously. It also provides a high resolving power for identification and interference-free detection of these gas species. The COPS system used in this study comprises an in-house developed and built Fourier transform spectrometer [36] combined with a mid-infrared supercontinuum source (SC-FTS). The supercontinuum (SC) source is a novel laser source with a high-power and low-noise ultrabroad spectrum in the mid-infrared spectral region (∼3 W, 2–11.5 μm) [42,43]. The SC source is water-cooled, and its laser head is purged with dry air. It consists of a mode-locked master oscillator and a single-pass power amplifier, both based on polycrystalline Cr:ZnS gain elements and optically pumped by Er-doped fiber lasers. The broad spectral coverage is produced using IDFG in a non-linear ZnGeP_2_ crystal. The source's unique, broad spectral range covers fundamental rovibrational bands of most small molecular gases (e.g., GHGs and polluting gases, as well as many other hydrocarbons, oxides, and small organic molecules). To the best of our knowledge, this is the first field deployment of such an ultra-broadband IDFG-based SC source out of a laboratory environment. The free-space output from the SC source is reflected by two off-axis parabolic mirrors (MPD129-P01 and MPD169-P01, Thorlabs) that expand the beam and optimize the collimation. The expanded beam is then sent over an open path for interaction with the targeted sample area. A 4.6-cm cubic retroreflector (HRR203-P01, Thorlabs) is used to reflect the beam and return it to the SC-FTS, where a second pair of off-axis parabolic mirrors (MPD249-P01 and MPD019-P01, Thorlabs) reduces the beam size and directs it to the FTS. The spectrum is measured every 2 s by the FTS with a 3 GHz (=0.1 cm^−1^) spectral resolution. The high spectral resolution of FTS resolves the absorption features at the natural atmospheric broadening, enabling optimal discrimination between gases with overlapping absorption features. The FTS is a Michelson-type interferometer that uses a balanced detection scheme to improve the signal-to-noise ratio (SNR) and enhance the detection sensitivity [36]. The entire system is integrated into a transportable 1.3 m × 1.3 m × 0.8 m cart.

### 2.2 Data collection

The FTS records an interferogram every 2 s, which is converted in post-processing to a power spectrum using fast Fourier transformation. Every power spectrum contains the entire spectral coverage of the source, meaning that all gas species absorbing the SC light are detected simultaneously. The power spectra are averaged for 40 s to further enhance the SNR and increase the sensitivity of the system. The averaged power spectra are transformed into absorbance spectra, so that the obtained absorption features scale linearly with the concentration of the molecules, following the Beer-Lambert law*.* This transformation is done by normalizing the power spectrum to a global baseline and a summation of sinusoidal functions to account for the general shape of the power spectrum and etalons introduced by the optics, most notably the beam splitter inside the FTS. Despite the beam splitter being wedged (Thorlabs BSW711), it is very common for FTS systems to have some residual etalon in the detected spectra that needs to be removed numerically. The HITRAN database [44] is used to construct reference spectra of the expected molecules (e.g., NH_3_, carbon monoxide (CO), N_2_O, CO_2_, CH_4_, and water (H_2_O)) in the environmental conditions during the measurement campaign (temperature: 17 ± 1 °C, atmospheric pressure 1013 ± 1 hPa). A fit of these reference spectra to the measurement spectra yields the concentration of each molecule integrated over the entire line-of-sight of the laser. Sinusoidal and polynomial reference spectra account for power spectrum fluctuations and etalons. The residual (the measured absorbance spectrum minus the concentration-scaled reference spectra) is minimized through this fitting; leaving no remaining absorption features indicates all gas species have been accounted for and their concentrations have been appropriately retrieved. A schematic overview of this data processing procedure is provided in the Supplementary Material Fig. S1.

### 2.3 The WWTP site and experimental protocol

**Fig. 1:**
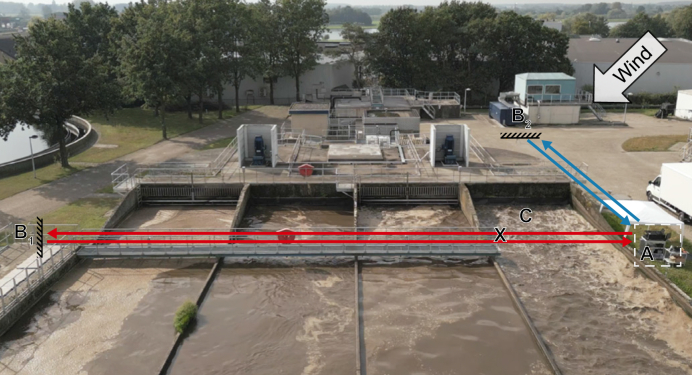
View of the aeration tank (AT) at the wastewater treatment plant with the coherent open-path spectroscopy system (A), retroreflectors (B_1_ and B_2_), the AT beam path (red), and upwind beam path (blue), both 60 m. The point measurement sampling location (X) is above the lane with the main aerator of the AT (C).

The measurements were performed above the aeration tank (AT) of the WWTP in Remmerden, the Netherlands (Fig. 1), in October 2023. The COPS system (A) was placed next to the AT, and the laser beam traveled towards a retroreflector (B_1_) over the AT and back to the spectrometer (red lines). To identify the background concentration of the gases, a second beam path was constructed perpendicular to the beam path over the AT and facing upwind (blue lines to B_2_). With a range finder (Coolshot Pro II, Nikon), both beam paths were verified to be 60.0 ± 0.8 m long. The COPS system was continuously monitoring the gases above the AT for approximately 4 h, after which the background concentrations were recorded for approximately 30 min.

During the COPS measurements, we employed several state-of-the-art commercial point measurement systems to measure the gases simultaneously retrieved by the COPS system. All these commercial systems use an internal gas cell where the gas is sampled from a location (marked X in Fig. 1) above the main aerator of the AT and along the beam path of the COPS system. CH_4_ and CO_2_ were measured using an off-axis integrated cavity output spectroscopy (OA-ICOS-based) system (Ultra-portable Greenhouse Gas Analyzer, ABB, Inc.). CO and N_2_O were measured using a mid-infrared laser-based system with a multipass cell (MIRA Ultra analyzer, Aeris Technologies, Inc.). NH_3_ was measured using a photoacoustic system (LSE NH_3_ Monitor 1700, LSE Monitors) equipped with a specially perfluoroalkoxy alkane (PFA)-coated cell and heated (∼40 °C) PFA tubing to minimize the adhesion of NH_3_.

The AT of the WWTP is a four-lane carrousel system equipped with two surface aerators to introduce oxygen to the water. The main aerator is located at the top of the right lane (C, Fig. 1). The AT is equipped with a probe to measure the dissolved oxygen (O_2_) concentration (LDO, Hach) and dissolved ammonium (NH_4_^+^) concentration (ANI-SE, Hach). Aeration is increased when the NH_4_^+^ concentration exceeds a threshold of ∼3.5 mg L^−1^.

## 3 Results and discussion

### 3.1 Determining detected molecular species and path-averaged concentrations

**Fig. 2:**
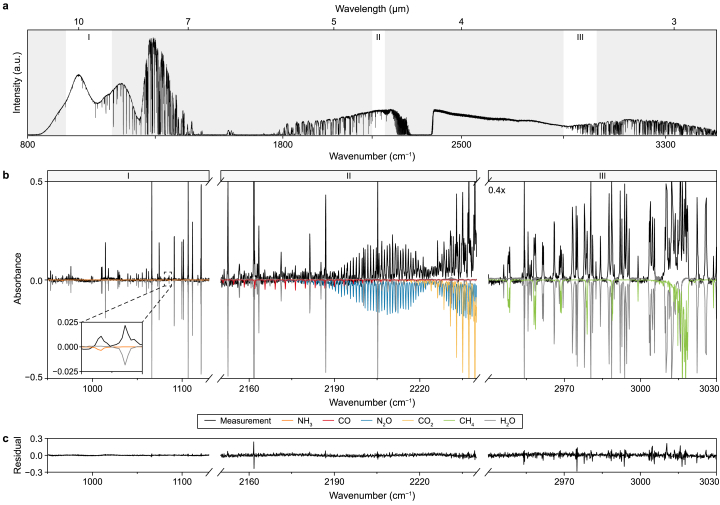
**a**–**b**, Power spectrum measured in 40 s over the aeration tank (**a**) with absorbance spectrum (**b**) zoomed to spectral ranges I, II, and III of the power spectrum for simultaneous detection of NH_3_, CO, N_2_O, CO_2_, CH_4_, and H_2_O. The absorbance spectrum is fitted to reference spectra (in color and inverted), and the right window is magnified 0.4 × to visualize relevant absorbance lines. **c**, The residual of the fit of reference spectra to the measured absorbance spectrum.

The 40-s averaged power spectrum of the measurement of a 60-m open path over the AT covering the entire wavelength region is shown in Fig. 2a. This is a significant part of the mid-IR fingerprint region related to the C–H stretching and C=O vibrational modes and, therefore, opens possibilities for the identification and sensitive detection of a great variety of gases [41]. The power spectrum predominantly contains transmission dips related to highly absorbing H_2_O and CO_2_ features. Relevant areas containing features of other gas species are marked white. The absorbance spectrum of these spectral ranges is shown in Fig. 2b, together with fitted, modeled reference spectra of the detected molecules (NH_3_, CO, N_2_O, CO_2_, CH_4_, and H_2_O). These spectra are shown in color and inverted for clarity. The fit of these reference spectra to the measured spectra yields the concentration of each molecule. The residual of this fit (Fig. 2c) demonstrates the high quality of the fitting since no visible absorption features are left, apart from a few remaining ones linked to highly absorbing (saturating) water lines. Therefore, these specific saturated water features were not considered when retrieving the water concentration, and they do not interfere with detecting other molecules. Thanks to the broadband spectrum, it is possible to select spectral regions with little water features or resolve features at a spectral resolution sufficient to discriminate the water features from the other molecules. Therefore, this demonstrates that the method is interference-free and able to resolve molecular concentrations without needing to calibrate or compensate for the water concentration.

**Fig. 3:**
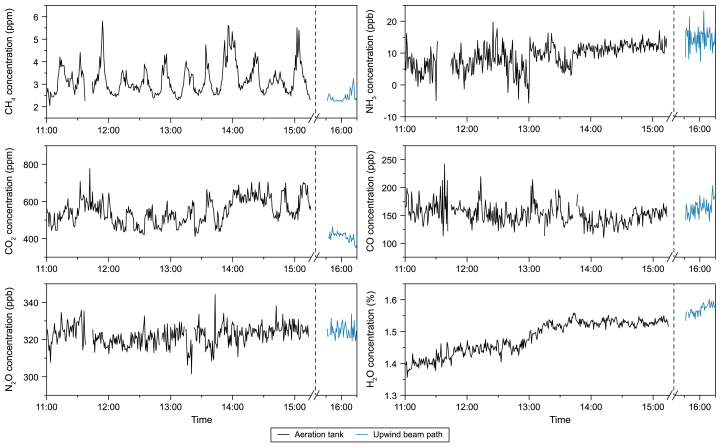
Path-averaged concentrations (40-s averages) of detected species over the aeration tank (black lines) and path-averaged background concentration over the upwind beam path (blue lines).

For each spectrum (i.e., every 40 s), the concentrations of the absorbing molecules detected by the COPS system are retrieved; their dynamics are displayed in Fig. 3.

The reported concentration is an average for the entire 60-m beam path, even though the concentration throughout the area might be unevenly distributed. The concentration might be higher in a plume from an emission source; this will be considered in section 3.2. The concentration can also be reported as the product of volume fraction and path length, which gives insights into the detection sensitivity of the system independent of the optical path length used. These values, which are called path-integrated concentrations, can be found in Supplementary Material Fig. S2. From 11:00 to 15:15, the COPS system measured over the AT (black line). For a shorter period, from 15:30 onwards, the system recorded the background concentrations upwind of the AT (blue line). In this period, the species detected and monitored over time were CH_4_, CO_2_, N_2_O, CO, H_2_O, and NH_3_. CH_4_ and CO_2_ have higher concentrations above the AT than the background concentrations and show a clear dynamic pattern. The concentrations of N_2_O, CO, NH_3_, and H_2_O are similar over the AT and the upwind beam path, retrieving typical concentrations of 329 ± 5 ppb, 157 ± 19 ppb, 11.9 ± 1.5 ppb, and 1.53 ± 0.01 %, respectively, that remain stable over time, confirming that there are no or only minor (detectable) emissions from the AT of these compounds over the considered measuring period. The increase in H_2_O relates to an increasing ambient temperature during the day.

The stable concentrations of N_2_O, CO, and NH_3_ measured in the upwind and the AT measurement path by the COPS system agree with the average concentrations detected by the point measurement devices (Table 1).

**Table 1:** Time-averaged concentrations of CO, NH_3_, and N_2_O measured above the aeration tank with the coherent open-path spectroscopy system (COPS) and the point measurement devices. The concentration was averaged over 1 h, with the standard deviation as uncertainty and a 40-s time interval per measurement point.

Molecule	Average concentration (COPS)	Average concentration (Point measurements)
CO	157 ± 19 ppb	165 ± 4 ppb
NH_3_	11.9 ± 1.5 ppb	11.6 ± 1.7 ppb
N_2_O	329 ± 5 ppb	333.2 ± 0.6 ppb

NH_3_ is detected at a concentration close to the detection limit of the COPS system*,* as indicated by the high noise in the retrieved concentrations (Fig. 3); however, it matches well with the concentration from the point-measurement instrument. Furthermore, these concentrations are in line with the literature on expected concentrations of these molecules in (semi-)urban environments [[45], [46], [47], [48]].

### 3.2 Estimating emission plume concentrations

**Fig. 4:**
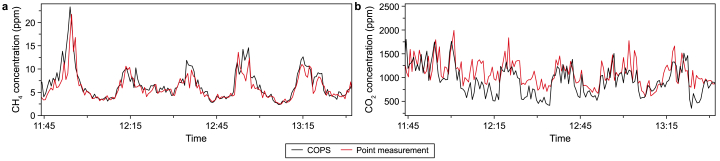
Plume concentrations of CH_4_ (**a**) and CO_2_ (**b**) at the aerator, measured and estimated with the coherent open-path spectroscopy system (COPS, black lines) and sampled from the plume with the OA-ICOS-based ABB point measurement system (red lines).

The temporal dynamics of the CH_4_ and CO_2_ concentrations observed with the COPS system (Fig. 3) indicate clear emission moments of these gases from the AT. Even though the COPS system monitors the integrated concentration over the entire line of sight of the beam, the concentration within the emission plume could be estimated. For comparison, the point measurement devices were placed specifically above the aeration point of the WWTP to sample from the emission plume and detect the concentration of these gases inside the plume. For the point measurements, it is crucial to sample at the right position in the plume, as the dynamic and elevated concentrations could not be observed at a different sampling point above the other lanes of the AT. The COPS system, however, does not depend on these strict limitations on the sampling location. To estimate the plume concentrations with the COPS system, the path-integrated concentration was considered for a path length of twice the width of the aeration channel (10 m), and atmospheric background concentrations (CH4: 2.3 ppm, CO2: 420 ppm) were subtracted for the remaining path length. The resulting concentrations show the same trend as those found by the point measurements (Fig. 4). The CH_4_ concentrations show a high level of correlation, while the CO_2_ concentrations demonstrate the same dynamics albeit at a slight offset between 12:00 and 12:45, possibly caused by diurnal fluctuations in the background CO_2_ level. Extended measurements of the background concentration in the upwind beam path would improve the accuracy of these plume concentration estimates. The correlations demonstrate that the COPS system can detect the concentrations within the emission plume with similar precision as a point measurement instrument held specifically at the emission point, even though the COPS system is observing its entire beam path at once (i.e. stand-off detection). Thus, the COPS system offers more flexibility in regions that can be monitored (as not all spots at the WWTP are easily accessible for point sampling), has a higher chance of detecting unforeseen emission points, and can detect multiple points simultaneously. Furthermore, retrieving a spatially-integrated concentration reduces the chance of errors due to spatial heterogeneity. In addition, the COPS system can simultaneously monitor many more molecular species than all the point measurement devices used in this study, combined.

### 3.3 Relation to WWTP operation

**Fig. 5:**
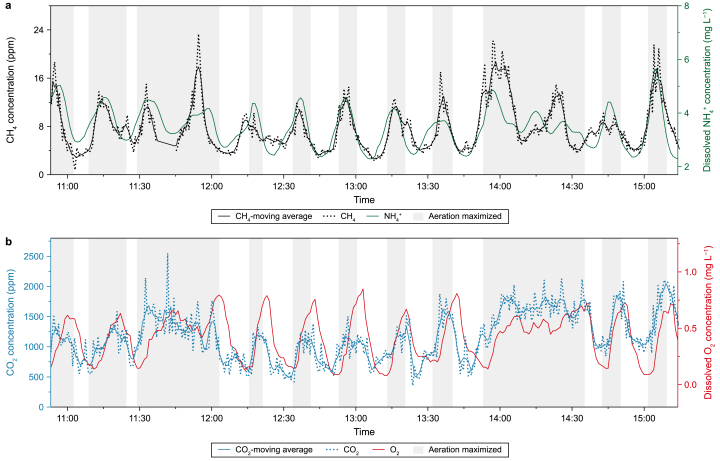
Plume concentrations of CH_4_ (**a**) and CO_2_ (**b**) above the aeration tank, measured by the coherent open-path spectroscopy system, with the periods of maximized aeration in grey shades and the NH_4_^+^ (**a**) and O_2_ (**b**) concentration.

In the AT of this specific WWTP, air is pushed through the wastewater during aeration using a surface aerator. The turbulence at the water-air interface enhances the exchange of dissolved CH_4_ and CO_2_ to the atmosphere. The operation of the aeration system is regulated by the measured dissolved ammonium (NH_4_^+^) concentration in the AT. NH_4_^+^ is an indicator of inflowing wastewater to the AT, as it is naturally present in domestic sewage and gets oxidized to nitrate (NO_3_^−^) by nitrifying microorganisms in the aerobic treatment process [49]. The detected gases above the AT can be related to the operation of the WWTP. In Fig. 5, the concentrations of CH_4_ and CO_2_ above the AT retrieved by the COPS system are shown with the periods in which the aeration is maximized (grey background) and the measured concentration of oxygen and NH_4_^+^ dissolved in the water. The CH_4_ and CO_2_ concentrations are the same as reported in Fig. 4 but smoothened using adjacent averaging to improve the clarity of the trends.

Both the emission of CO_2_ and CH_4_ can be linked to maximal system aeration (Fig. 5). The concentrations of CH_4_ and NH_4_^+^ (black and green lines) follow the same trend, indicating that the CH_4_ emissions peak when wastewater flows into the AT. CH_4_ is produced by methanogenic archaea as a product of the anaerobic conversion of the fermentation products acetate and hydrogen within the sewage and the pipelines of the wastewater treatment process before entering the AT [9,50,51]. Aeration enhances the oxygen concentration in the wastewater for the aerobic nitrification of NH_4_^+^ to NO_3_^−^, which can then be converted to nitrogen gas (N_2_) under oxygen-limited conditions. Similarly, aerobic processes convert organic carbon-rich compounds into CO_2_, while anaerobic processes can convert these compounds to CH_4_ in addition to (some) CO_2_ [52]. Therefore, the similar dynamics between gaseous CO_2_ and dissolved O_2_ (blue and red lines) can be well explained. During every aeration period, O_2_ is brought into the wastewater. The dissolved O_2_ rapidly decreases when the aeration is tuned down. The remaining O_2_ is converted to CO_2_ that dissolves well in water. When the aeration is turned on again, CO_2_ emission peaks.

### 3.4 Implications

The broadband coverage and the high spectral resolution of the COPS system enable a high resolving power for identification and interference-free detection of an extensive number of gas species. While CH_4_, CO_2_, N_2_O, NH_3_, CO, and H_2_O were monitored at the WWTP, these gases do not represent an exhaustive list of detectable species. In previous work, it has been demonstrated that the COPS system can detect, among other molecules, hydrocarbons, oxides, and small organic molecules in a laboratory setting [42]. Simultaneous measurements of more (trace) gases and pollutants enable a deep understanding of biogeochemical processes and validation of multi-scale models.

Although N_2_O was monitored during this study, no sustained emissions have been found. The stable, matching concentrations between the AT and upwind beam path indicate that the system successfully detects this greenhouse gas and the AT did not emit considerable amounts of this gas at the time of measurement. N_2_O emissions from WWTPs are of high interest due to the limited available information on the total emission of this strong GHG from the WWTP, as well as its variability over time. The emissions are known to be related to incomplete (de)nitrification and have a strong temporal dynamic, showing to be spiking at specific moments, such as during rapidly changing processes or environmental conditions [1]. For this reason, having measurement solutions that can continuously monitor areas is instrumental and a major advantage over labor-intensive discrete sampling approaches. The COPS system is a well-suited candidate for such continuous measurement campaigns. The system can operate autonomously, resulting in an efficient, cost-effective solution for emission monitoring and characterization over prolonged periods, including day-night cycles and seasonal variations.

A clear benefit of the COPS system over point-measurement systems is the ability to detect these emissions without carefully searching and moving the system to different positions to find the emission location/area. Incorrect positioning of point measurement systems can cause over- or underestimation of gas concentrations emitted from an unexpected or inhomogeneous source. By scanning over an open path, a bigger area can be monitored simultaneously, overcoming the spatial limitations of point measurement techniques in heterogeneous environments. Future refinements of the technique will allow higher sampling rates that would facilitate combined analysis of gas concentration changes and atmospheric mass flow [53]. Furthermore, as the system does not need to be moved, it is also easier to keep track of temporal changes in the emission rates. Finally, another advantage of COPS is its calibration-free methodology, as well as the fact that no gas sample treatment is needed (i.e., preventing wall-adhesions effects in sampling NH_3_).

## 4 Conclusions and outlook

The uniquely broad spectral coverage of the SC source in the COPS system (2–11.5 μm) in combination with ∼3 W power enables the simultaneous detection of a wide variety of molecular gases with a high sensitivity and a high dynamic range. For molecules with stable atmospheric concentrations (CO, NH_3_, and N_2_O), estimated sensitivities were reached of 19, 1.5, and 5 ppb in 40 s, respectively. This novel *COPS* system has significantly broader spectral coverage than current dual-comb systems [28,32], increasing the scope of detectable molecular species and the dynamic range due to the high number of detectable absorption lines [42].

We demonstrated the first field deployment of such a system. Over an open path, gas concentrations were monitored above and upwind of the AT of a WWTP. Several species, such as CH_4_, CO_2_, N_2_O, NH_3_, CO, and H_2_O, were present at detectable concentrations at the WWTP. The temporal dynamics and elevated concentrations of CH_4_ and CO_2_ indicated clear emissions of these compounds from the AT. Similar dynamics were observed using commercial laser-based point-sampling spectroscopy instruments. These temporal dynamics link to the operation of the WWTP, showing the relation of CO_2_ and CH_4_ emissions to the inflow of wastewater to the AT, aerobic processes in the AT, and the aerator speed.

The system successfully detected N_2_O, CO, and NH_3_ in the measurements of the beam path over the AT and the upwind beam path. We conclude that the AT did not emit considerable amounts of these gases at the time of measurement, as stable, matching concentrations were found between both paths.

The results presented here demonstrate the COPS technology in a field application at a WWTP. This represents the first step towards real-time emission monitoring and quantification of GHG and other gases relevant to complementing the nitrogen and carbon balances of the WWTPs. In particular, quantifying these emissions over extended periods, ranging from day–night cycles to varying weather conditions or seasonal variations, will provide valuable data to WWTP operators for more accurate annual GHG budgets and better decision-making on mitigation strategies to ensure WWTP sustainability.

## CRediT authorship contribution statement

**Roderik Krebbers:** Writing – review & editing, Writing – original draft, Visualization, Validation, Supervision, Software, Resources, Project administration, Methodology, Investigation, Formal analysis, Data curation, Conceptualization. **Kees van Kempen:** Writing – review & editing, Validation, Software, Resources, Methodology, Investigation, Data curation, Conceptualization. **Yueyu Lin:** Writing – review & editing, Validation, Investigation, Formal analysis. **Joris Meurs:** Writing – review & editing, Visualization, Methodology, Formal analysis. **Lisanne Hendriks:** Writing – review & editing, Validation, Resources, Methodology, Data curation, Conceptualization. **Ralf Aben:** Writing – review & editing, Writing – original draft, Visualization, Validation, Resources, Methodology, Conceptualization. **José R. Paranaiba:** Writing – review & editing, Writing – original draft, Validation, Resources, Methodology, Data curation, Conceptualization. **Christian Fritz:** Writing – review & editing, Methodology, Conceptualization. **Annelies J. Veraart:** Writing – review & editing, Writing – original draft, Supervision, Resources, Methodology, Funding acquisition, Conceptualization. **Amir Khodabakhsh:** Writing – review & editing, Validation, Supervision, Software, Project administration, Methodology, Investigation, Funding acquisition, Formal analysis, Conceptualization. **Simona M. Cristescu:** Writing – review & editing, Visualization, Validation, Supervision, Project administration, Methodology, Investigation, Funding acquisition, Formal analysis, Conceptualization.

## Declaration of interests

The authors declare that they have no known competing financial interests or personal relationships that could have appeared to influence the work reported in this paper.
