# Supplementary material for: Ultra-broadband coherent open-path spectroscopy for multi-gas monitoring in wastewater treatment
Source: Environ Sci Ecotechnol. 2025 Mar 17;25:100554. doi: 10.1016/j.ese.2025.100554 (PMC11987694; doi:10.1016/j.ese.2025.100554)
Supplement: Multimedia component 1 [file mmc1.pdf]

Supporting materials for

# Ultra-Broadband Coherent Open-Path Spectroscopy for Multi-Gas Monitoring in Wastewater Treatment

Roderik Krebbers[a], Kees van Kempena[a], Yueyu Lin[a], Joris Meurs[a], Lisanne Hendriks[b], Ralf Aben[b], José R. Paranaiba[b], Christian Fritz[b], Annelies J. Veraart[b], Amir Khodabakhsh[a], Simona M. Cristescu[a*]

a. Life Science Trace Detection Laboratory, Department of Analytical Chemistry & Chemometrics, Institute for Molecules and Materials, Radboud University, Heyendaalseweg 135, 6525 AJ Nijmegen, The Netherlands

b. Department of Ecology, Radboud Institute for Biological and Environmental Science, Radboud University, Heyendaalseweg 135, 6525 AJ Nijmegen, The Netherlands

* Corresponding author. Email: simona.cristescu@ru.nl

**Summary of Contents:**

Figure S1

Figure S2

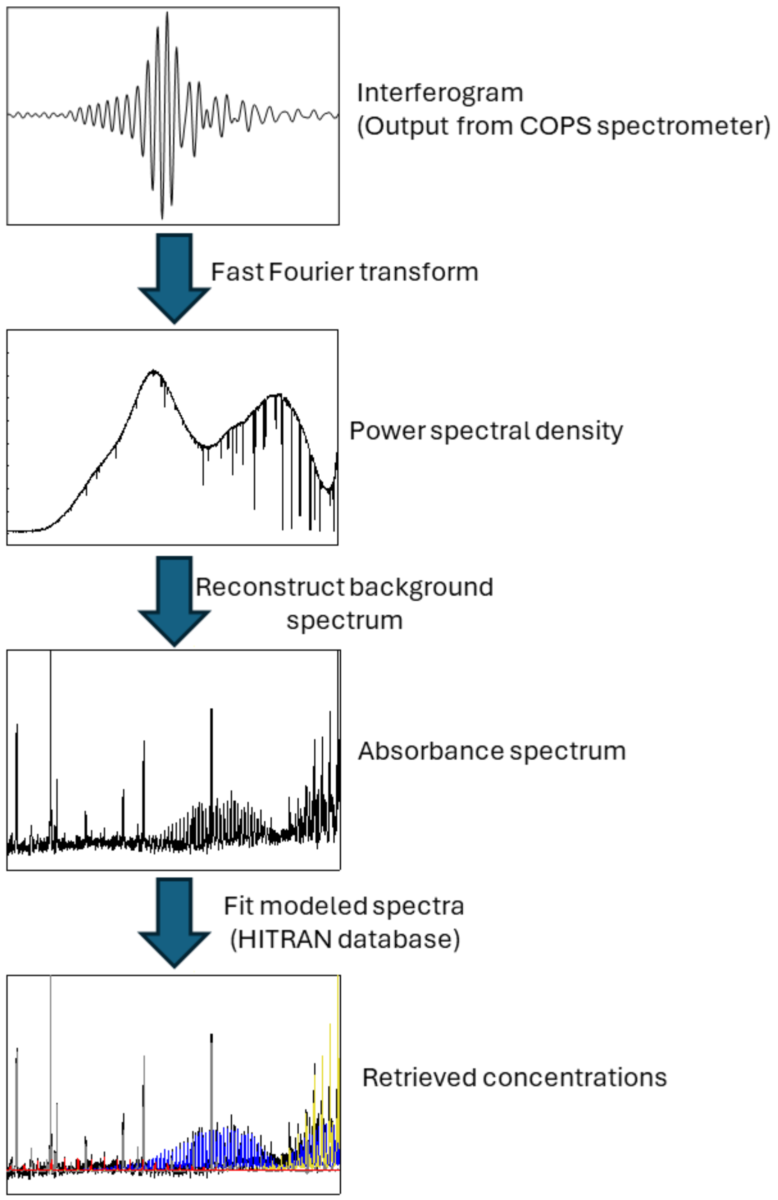


**Figure S1.** Schematic overview of the data processing procedure.

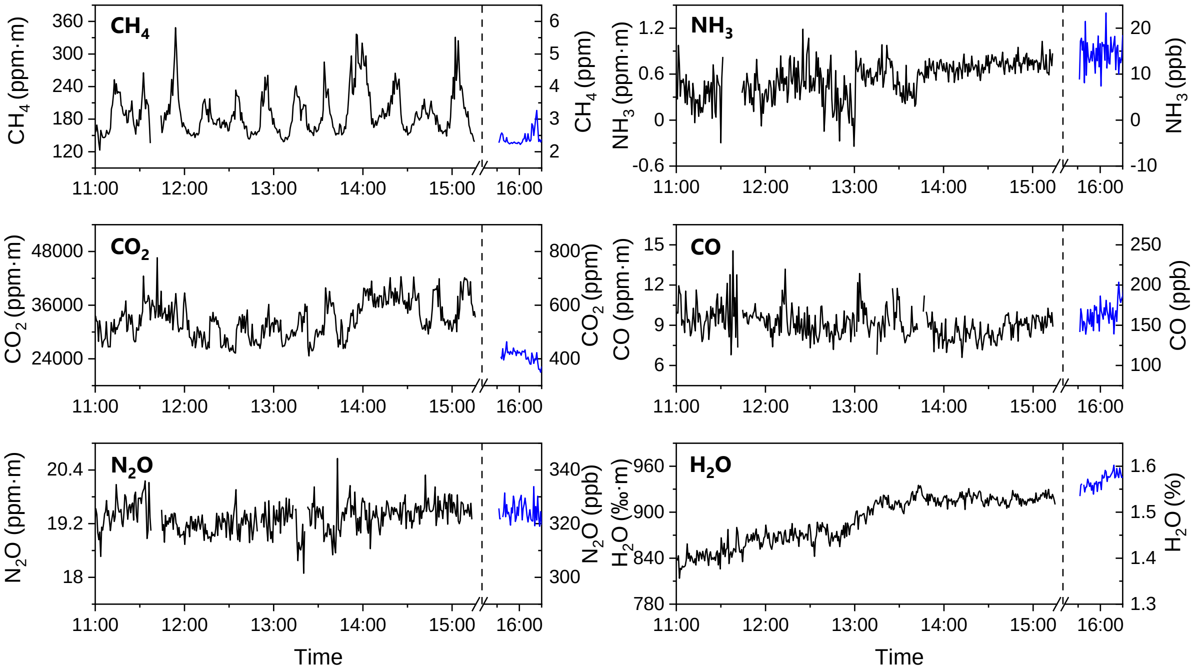


**Figure S2.** Path-integrated concentrations (40-second averages) of detected species over the AT (in black) and path-integrated background concentration over the upwind beam path (in blue).

The reported concentration in Figure 3 is an average for the entire 60-meter beam path, even though the concentration throughout the area might be unevenly distributed. As the absorbance strength of the gas species scales linearly with both the path length and concentration, it is also possible to report path-integrated concentrations as the product of volume fraction and path length instead of the volume fraction only. This gives insights into the detection sensitivity of the system, independent of the optical path length used. Therefore, Figure S2 reports path-integrated concentrations (in units of the product of volume fraction and path length, left axis) as well as the path-averaged concentrations (in units of volume fraction, right axis).
